# The Effect of Behavioral Intervention on Maternal Breastfeeding Practice and Infant Growth in Congenital Heart Disease: A Randomized Controlled Trial

**DOI:** 10.1002/fsn3.70907

**Published:** 2025-09-14

**Authors:** Huimei Wang, Qi Zhang, Yu Sun, Xueping Zhang, Yuehong Ren, Yalan Dou, Chuyi Zhan, Ming Ye, Ying Gu

**Affiliations:** ^1^ Department of Cardiac Surgery Children's Hospital of Fudan University Shanghai China; ^2^ School of Nursing Fudan University Shanghai China; ^3^ Department of NICU Children's Hospital of Fudan University Shanghai China; ^4^ Department of Clinical Trial Unit Children's Hospital of Fudan University Shanghai China; ^5^ Nursing Department Children's Hospital of Fudan University Shanghai China

**Keywords:** behavioral intervention, breastfeeding, congenital heart disease, exclusive breastfeeding

## Abstract

Breastfeeding offers critical health benefits for infants, including those with mild congenital heart disease (CHD). However, breastfeeding in this population faces multifaceted challenges. At the individual level, maternal anxiety associated with the CHD diagnosis may undermine breastfeeding confidence; at the systemic level, institutional support for establishing and sustaining breastfeeding remains inadequate. Behavioral interventions may influence feeding practices for these infants. To evaluate the effect of a Behavioral Breastfeeding Intervention Program (BBIP) for infants with mild CHD on maternal breastfeeding behavior, exclusive breastfeeding (EBF) rates, and infant growth. Sixty‐eight mother‐infant dyads were randomly assigned to the BBIP group (*n* = 34) or a control group receiving routine care (*n* = 34). The BBIP was grounded in the Behavior Change Wheel (BCW) behavior change theory and included personalized counseling, home visits, and ongoing support via social media. The primary outcome was breastfeeding behavioral scores. Secondary outcomes included EBF rates, the proportion of breast milk in the daily diet, and infant growth metrics assessed at 1, 3, and 6 months. Mothers in the BBIP group reported significantly higher breastfeeding behavioral scores (*p* < 0.001). EBF rates at 1, 3, and 6 months were 47.1%, 45.5%, and 43.8%. Proportion of breast milk in the daily diet was approximately 78.35%, 69.67%, and 56.40%. Growth data over 6 months showed non‐inferiority in outcomes for infants with CHD. The behavioral intervention significantly enhanced breastfeeding practices among mothers of infants with mild CHD through multidimensional strategies. Non‐inferior growth trajectories were observed in breastfed infants.

## Graphical abstract

The Behavioral Breastfeeding Intervention Program (BBIP), based on the Behavior Change Wheel (BCW) theory and delivered via personalized counseling, home visits, and social media support, significantly improved breastfeeding practices among mothers of infants with mild CHD. Throughout the 6‐month intervention, exclusive breastfeeding rates and the proportion of breast milk in infants' daily diets showed significant improvement. Breastfed infants with mild CHD demonstrated non‐inferior growth outcomes.

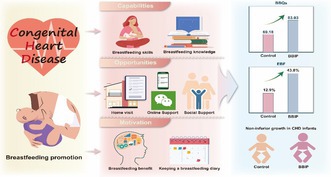

## 1 Introduction

Congenital heart disease (CHD) is one of the most common congenital defects, with considerable inter‐regional variability in prevalence. The estimated mean prevalence of CHD globally is 8.224 per thousand (Liu et al. 2019). The widespread implementation of CHD screening in China has helped increase the number of CHD cases diagnosed at birth. In 2020, the estimated prevalence of CHD in China 2020 was 17.32 cases per 1000 perinatal births (Zhang et al. 2023). The prevalence of mild CHD (Hoffman and Kaplan 2002) witnessed an approximately threefold increase from 1970 to 2017, accounting for 70%–80% of the total number of CHD cases (Giang et al. 2023; Lucron et al. 2024). Developmental disorders and disabilities encompass a broad range of developmental delays or abnormalities and are increasingly recognized as a common outcome of CHD (Asschenfeldt et al. 2020; Marino et al. 2012).

Breastfeeding plays a crucial role in promoting the physical and cognitive development of children (Wallenborn et al. 2021). Human milk feedings reduce the risk of necrotizing enterocolitis in premature infants. Direct breastfeeding minimizes oxygen consumption and energy expenditure (Blanco et al. 2023; Cognata et al. 2019; Elgersma et al. 2023). The 2023 Science Advisory from the American Heart Association emphasizes human milk and breastfeeding as being essential to the developmental care of infants with critical CHD (Lisanti et al. 2023). Despite the numerous benefits of breastfeeding, breastfeeding rates among infants with CHD remain suboptimal. Several variables may influence the maintenance of breastfeeding in infants with CHD, including lesion severity, the requirement for mechanical ventilation or early surgery, breathing difficulties, and heart failure, as well as psychological and social factors (Agostini et al. 2021; Elgersma et al. 2023; Goulart et al. 2020).

The World Health Assembly aims to increase the rate of exclusive breastfeeding in the first 6 months to 50% by 2025 (World Health Organization 2014). Even with complementary foods, continued breastfeeding up to 2 years of age is recommended (Victora et al. 2016). Studies indicate that among CHD infants undergoing cardiac surgery, the exclusive breastfeeding rate at 6 months is only 31% (with any breastfeeding rate at 57%) (Torowicz et al. 2015). A cross‐sectional study from Brazil reported that a mere 15.7% of CHD infants achieved exclusive breastfeeding (Goulart et al. 2020), a rate substantially lower than the norm. In China, exclusive breastfeeding rates among healthy infants increased from 36.69% to 47.9% within the first 6 months between 2013 and 2018; significant regional disparities persist (Li et al. 2023). This proportion may be even lower in children with CHD.

Breastfeeding challenges for infants with CHD arise from various factors, including maternal–infant separation due to medical interventions, inadequate maternal knowledge and confidence in breastfeeding, and lack of support from family and medical teams (Davis and Spatz 2019; Russel et al. 2024). Adequate maternal support has been shown to improve breastfeeding outcomes (Al Ghazal et al. 2015; Ruiz et al. 2025). Research highlights the multifaceted needs of breastfeeding mothers, which encompass enhancing their information and skills, increasing opportunities, improving reflexes and motivation, and creating supportive environments (Hamnøy et al. 2024). Behavioral interventions have been shown to positively impact maternal mental health and breastfeeding outcomes (Pezley et al. 2022).

The Behavior Change Wheel (BCW), developed by Michie et al. (2011), integrates 19 theoretical frameworks for behavioral intervention. This comprehensive framework enables researchers to identify and analyze the underlying sources and influencing factors of target behaviors. By considering the complex interplay of relevant factors, the BCW also examines how the external environment influences target behaviors, thereby maximizing the potential for individual behavior change. This theory has been extensively applied for individual health promotion, chronic disease management, and mental health enhancement (Faija et al. 2021; Reid et al. 2022). The BCW framework can help identify and categorize various determinants of breastfeeding behavior, enabling the development of more targeted intervention strategies (Musgrave et al. 2021).

The relationship between breastfeeding and the outcomes of infants with CHD is not well characterized. This study aims to develop a BCW theory‐based behavioral intervention and evaluate its effects on maternal feeding behaviors and infant growth outcomes in children with CHD. We hypothesize that the behavioral intervention based on the BCW theory will increase breastfeeding rates and prolong breastfeeding duration among mothers of infants with CHD, thereby promoting better growth outcomes in these infants.

## 2 Materials and Methods

### 2.1 Study Design and Setting

This was a single‐center, parallel‐randomized controlled study. Mothers of neonates diagnosed with CHD were randomly assigned to the intervention group or control group (1:1 ratio) between April 2023 and August 2023 at the cardiac outpatient clinic of a children's hospital in Shanghai. The trial was approved by the Ethics Review Committee of the children's hospital (ethical number: 2022–375) and was registered on ClinicalTrials.gov (NCT: 05961540).

### 2.2 Participants

The inclusion criteria were as follows: (1) infants diagnosed with mild CHD (VSD, ASD, PDA, PS) within 14 days of birth, with exclusive breastfeeding at the time of enrollment and having mothers as the primary caregivers; (2) mothers owned a smartphone and had the skills to use it; (3) mothers were in good physical health; (4) age ≥ 18 years. The exclusion criteria were as follows: (1) infants with other comorbid conditions (congenital digestive tract malformations; facial malformations, such as cleft lip and cleft palate; various syndromes caused by chromosomal abnormalities, etc.); (2) infants with complex heart disease requiring cardiac surgical intervention within 14 days of birth; (3) mothers with contraindications to breastfeeding; (4) mothers with psychiatric disorders, cognitive disorders, and other conditions that can prevent normal communication.

In this study, breastfeeding definitions adhere to the WHO classifications, breastfeeding by a wet nurse, feeding of expressed breast milk, and feeding of donor human milk all count as being fed breast milk. Exclusive breastfeeding is defined as breastfeeding with no other food or drink, not even water.

### 2.3 Sample Size

**FIGURE 1:**
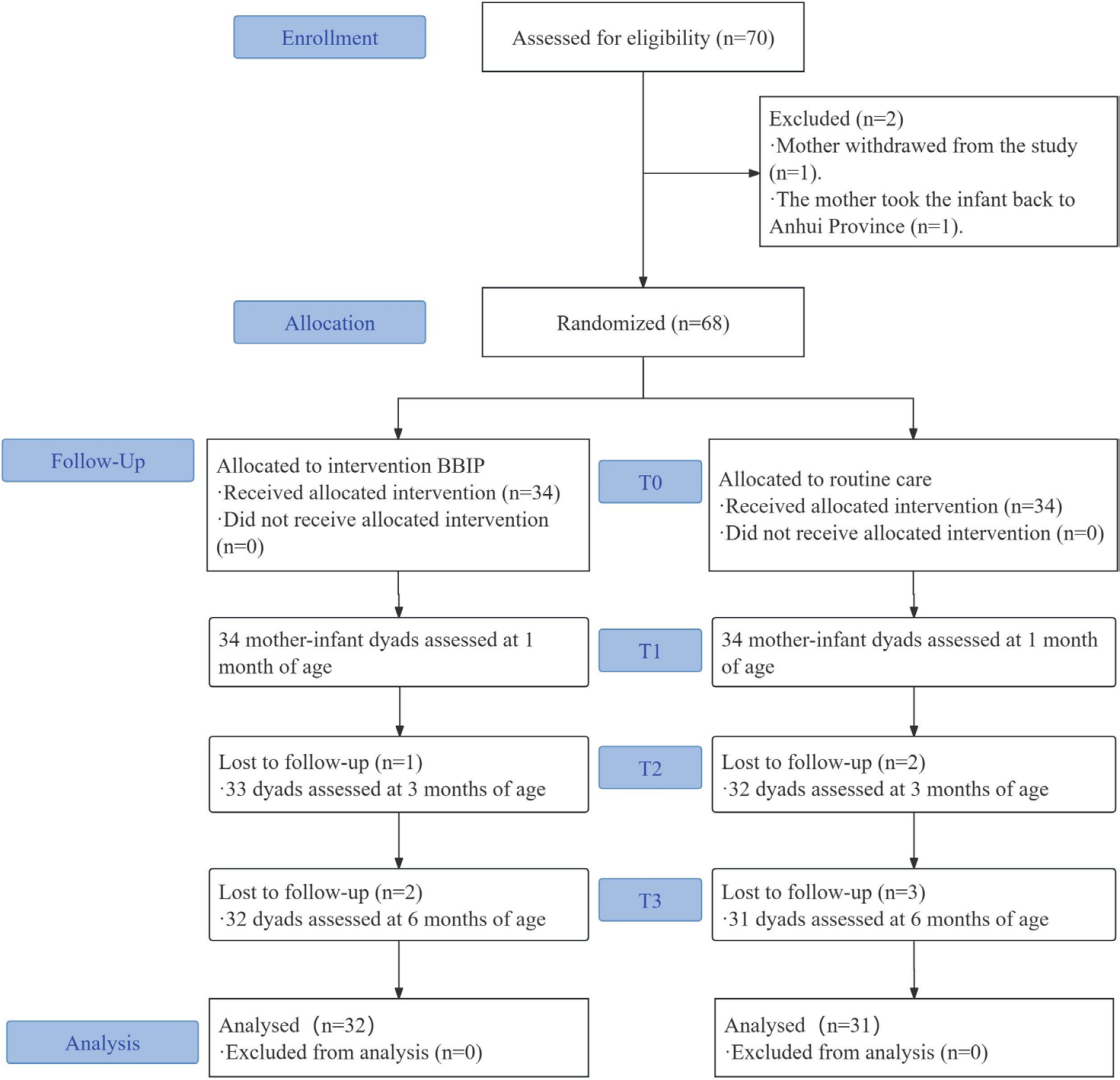
Flow chart detailing the number of participants selected, response rates, and attrition.

The sample size was calculated based on the changes in the maternal feeding behavior questionnaire scores. According to our preliminary pilot study, the mean change in questionnaire scores in the routine care and BBIP groups was 1.1 ± 9.8 and 13.8 ± 13.5, respectively. Setting the power at 0.9 and factoring two‐sided α = 0.05, calculations were performed using PASS 15 software. The required sample size was 31 CHD mother‐infant dyads per group. Factoring a 10% attrition rate, 34 dyads were recruited per group, for a total of 68 participants. The details of the number of participants, response rates, and attrition are presented in a flowchart (Figure 1).

### 2.4 Randomization

Participants were recruited from the cardiology outpatient clinic. The basic information of patients was obtained through the CHD screening referral platform and communication with the mothers to ensure that they qualified for the inclusion criteria and were willing to participate in the study. A randomized block design was used to ensure a balanced representation of the participants in the two groups (block length: 4; number of blocks: 17). The sequence was determined using computer‐generated random numbers. The generated sequences were numbered and placed in sealed opaque envelopes with corresponding numbers. A nurse not involved in the intervention was responsible for opening the envelopes in the numbered sequence to determine the group allocation. The research team members enrolled CHD mother‐infant dyads through the envelope sequence. All participants signed a written informed consent before enrollment, and the consent process was carried out by the project investigator, which included a verbal explanation of the study objectives, research plan, risks and benefits, and confidentiality principles, and provision of a paper version of the consent form. This study implemented blinding of outcome assessors.

### 2.5 Intervention

This study was based on the BCW theory, which aims to interactively alter behavior by cultivating and enhancing individual capabilities, opportunities, and motivation. This study was a nurse‐led study. All participating researchers have completed the training related to the project. The primary research team members have completed the International Board Certified Lactation Consultant (IBCLC) course and possess expertise in pediatric cardiac nursing. The study's primary interventions included home visits, in‐person breastfeeding guidance, targeted information provision, and personalized one‐on‐one support workgroups to facilitate successful breastfeeding.

The Behavioral Breastfeeding Intervention Program (BBIP) for mothers of infants with CHD, based on the BCW theory, included the following components:

1. At the first outpatient cardiac ultrasound examination of the newborn, the cardiology outpatient clinic nurses randomly assigned the participants to the group and established a WeChat group. The research nurse then conducted the initial face‐to‐face communication with the mothers to understand the current status of breastfeeding. The WeChat group for participants in this study was one‐to‐one and included the patient's main family members, the child's father and mother, in addition to two research nurses, one breastfeeding trainer, and one project coordinator.
2. Within 3 days of enrollment in the intervention group, the research team members conducted home visits, providing one‐on‐one guidance to the mothers and primary family members, lasting approximately 40–60 min. ① During structured home visits, IBCLCs educated mothers on the clinical benefits of breastfeeding for infants with CHD, demonstrating evidence‐based lactation enhancement skills, including breast massage, manual expression, and mastitis management protocols, while instructing on optimal feeding positions; they further facilitated family support through targeted caregiver communication to reinforce a positive feeding environment. ② Cardiac nurses trained mothers to identify critical feeding‐related warning signs in CHD infants, including choking episodes, prolonged feeding duration (> 30 min), profuse sweating, and peri‐oral cyanosis. ③ Standardized feeding documentation was implemented by instructing mothers and caregivers to maintain detailed diaries recording single‐feed volumes and 24‐h total milk intake. ④ For mothers utilizing breast pumps, hands‐on training covered medical‐grade pump operation and evidence‐based milk storage protocols featuring layered freezing techniques with strict time–temperature labeling. ⑤ For direct breastfeeding, mothers received standardized training in calibrated pre/post‐feed weighing procedures, requiring consistent infant clothing or diaper conditions and immediate weight differential conversion (1 g = 1 mL milk) using clinical‐grade digital scales.
3. Within 7 days following the home visit, the research nurse engaged with mothers through the dedicated WeChat group regarding breastfeeding status and its continuation. Researchers provided individualized breastfeeding support through WeChat groups, disseminating tailored educational content based on maternal needs.
4. Throughout the 6‐month enrollment period, the research nurse provided ongoing support to mothers through online query resolution, diary maintenance encouragement, and timely reminders for follow‐up appointments. Additionally, targeted home visits were conducted for families requiring extra support.

In the control group, CHD mother‐infant dyads received standard care at the Cardiovascular Center of a children's hospital in Shanghai. At the initial cardiac ultrasound screening visit, outpatient nurses educated parents on the benefits of breastfeeding and provided a feeding guidebook for infants with CHD. Parents were also instructed to attend scheduled follow‐up visits and were provided with disease‐specific knowledge. Both groups used identical feeding guidebooks.

Intervention fidelity was ensured through a standardized protocol and comprehensive training for all team members. The Coordinator monitored adherence via documentation review, direct observation using structured checklists. Data accuracy was ensured through dual independent entry with cross‐verification.

### 2.6 Measurements

The primary outcome measure was the maternal breastfeeding behavior questionnaire scores for mothers of infants with CHD administered at 1, 3, and 6 months of age. Secondary outcomes included EBF rates, the proportion of breast milk in the daily diet, and infant growth metrics assessed at the same time points. The maternal breastfeeding behavior questionnaire was administered initially in person and subsequently online. Infant growth metrics were initially measured by researchers during home visits and later by community pediatricians during routine community health check‐ups.

#### 2.6.1 Clinical Data

Clinical data included maternal age, conception method, gestational age, educational level, occupation, and income. Data for CHD infants included disease type, physical growth indicators (weight, length, developmental screening result from monthly checkup), feeding information (number of breastfeeds per day, daily breastfeeding intake, total daily intake). The weight and length data were converted to weight‐for‐age Z score (WAZ) and length‐for‐age Z score (LAZ) according to the Weight Score Calculator and the Length Score Calculator for Infants 0–2 years of Age provided by UpToDate. This data was extracted from medical records, telephone follow‐ups, and online questionnaires.

#### 2.6.2 Maternal Feeding Behavior

Based on the theoretical framework of the BCW, this study initially constructed a questionnaire covering five dimensions: breastfeeding attention, cues recognition, breastfeeding skills, lactation promotion, and abnormality recognition through literature review. The research team first conducted semi‐structured interviews with 12 mothers of CHD children to improve the item description, and then formed a multidisciplinary expert team (including eight experts in nutrition, breastfeeding, clinical medicine, and nursing) to conduct two rounds of Delphi method demonstration, and finally formed a formal questionnaire containing 21 items. The tool includes five dimensions: breastfeeding attention (3 items/15 points), cues recognition (4 items/20 points), breastfeeding skills (4 items/20 points), lactation promotion (6 items/30 points), abnormality recognition (4 items/20 points), with a total score range of 21–105 points. After evaluation by seven experts in the field, the content validity index of the questionnaire reached the optimal level (S‐CVI/UA = 1.00, S‐CVI/Ave = 1.00). In the pre‐experimental phase, the Breastfeeding Behavior Questionnaire (BBQ) demonstrated high internal consistency (Cronbach's *α* = 0.856) among 30 breastfeeding mothers of CHD infants, leading to the finalized version through item refinement and content validation.

#### 2.6.3 Infant Anthropometrics

A standardized high‐precision measuring instrument was used to collect infant anthropometric data, with length measurements accurate to 0.1 cm and weight measurements accurate to 0.01 kg.

### 2.7 Data Collection

Data on CHD infants, including growth information, were collected through telephone follow‐ups at 1, 3, and 6 months of age. Daily breast milk intake volumes were documented by mothers in breastfeeding logs. Research staff conducted weekly follow‐ups via telephone or WeChat to verify recording completeness. Infant growth parameters were initially measured by research staff during clinic visits and home assessments, with subsequent monitoring conducted by community pediatricians during routine well‐child visits.

### 2.8 Statistical Analysis

Data were analyzed using IBM SPSS Statistics 27.0. Categorical variables were expressed as rates and constitutive ratios, and continuous variables were expressed as means ± standard deviations if normally distributed. Between‐group differences were assessed for statistical significance using two independent samples *t*‐tests, chi‐square tests, or rank‐sum tests. A mixed‐effects model was employed to compare outcome indicators between the BBIP group and the routine care group, accommodating missing data. This approach allowed for flexibility in exploring a variety of possible trends, accounting for temporal and individual variations within groups, providing a comprehensive understanding of the overall effect. Dichotomous variables, such as exclusive breastfeeding at each month of age, were analyzed using a generalized mixed‐effects model. Breastfeeding as a percentage of total feeds (%) and WAZ and LAZ at 1, 3, and 6 months of age were treated as continuous variables and analyzed using a linear mixed‐effects model; this model analyzed the group effect, the time effect, and the group × time interaction. Subgroup and time effects of the interactions were included in the final model if statistically significant (*p* < 0.05) or not reported in detail.

## 3 Results

### 3.1 Baseline Characteristics

A total of 68 CHD infants and their mothers were recruited, and 63 completed the trial (BBIP group = 32, routine care group = 31). Five dyads dropped out because of being unreachable or their refusal to provide information. The baseline characteristics of the participants in the two groups were comparable (Table 1).

**TABLE 1:** Baseline demographic and clinical characteristics.

Project	BBIP (*n* = 34)	Routine care (*n* = 34)	Test statistic	*p*
Sex [*n*, %]			0.530^b^	0.467
Male	15 (44.12)	18 (52.94)		
Female	19 (55.88)	16 (47.06)		
Weeks of gestation [*n*, %]			0.000^b^	1.000
Full term	32 (94.12)	31 (91.18)		
Preterm	2 (5.88)	3 (8.82)		
Mode of birth [*n*, %]			0.243^b^	0.622
Normal delivery	13 (38.24)	15 (44.12)		
Cesarean section	21 (61.76)	19 (55.88)		
Birth weight [kg]	3.42 ± 0.43	3.27 ± 0.54	−1.268^a^	0.209
Birth length [cm]	49.85 ± 1.15	49.74 ± 1.38	−0.353^a^	0.725
Disease Type [*n*, %]			1.836^b^	0.766
ASD	9	6		
VSD	7	9		
PDA	5	5		
VSD + ASD/+PH/+PDA	5	3		
PS	8	11		
LVEF [%]	69.62 ± 5.68	70.62 ± 4.81	0.784^a^	0.436
Mother's education level [*n*, %]			−0.958^b^	0.338
Junior high school and below	2 (5.88)	3 (8.82)		
High school/secondary school	2 (5.88)	4 (11.76)		
College and above	30 (88.24)	27 (79.41)		
Maternal age [years]	30.62 ± 2.92	30.00 ± 2.88	−0.877^a^	0.383
Feeding food [%]			1.619^b^	0.203
Breastfeeding	8 (23.53)	4 (11.76)		
Mixed feeding	26 (76.47)	30 (88.24)		
Total daily intake [mL]	563.47 ± 164.07	503.91 ± 132.41	−1.647^a^	0.104

*Note:* The higher proportion of mixed feeding at baseline may attributable to data collection occurring within 7 days post‐enrollment, during which feeding practices may have changed.

Abbreviations: ASD, atrial septal defect; LVEF, left ventricular ejection fraction; PDA, patent ductus arteriosus; PH, pulmonary hypertension; PS, pulmonary stenosis; VSD, ventricular septal defect.

^a^ *t*‐value.

^b^ *χ* ^2^‐value.

### 3.2 Breastfeeding Behavior Questionnaire Scores (BBQS) of Mothers

At the first post‐intervention month (T1), the BBQS in the BBIP group increased from 69.18 (95% CI, 65.55, 72.80) to 83.03 (95% CI, 80.04, 86.02). During T2 and T3 follow‐ups, scores remained stable and were significantly higher than controls (*p* < 0.001). (Table 2).

**TABLE 2:** Breastfeeding behavior questionnaire scores in two groups.

Time	Breastfeeding behavior score				*t*	*p*
	BBIP (*n* = 34)	95% CI (LL, UL)	Routine care (*n* = 34)	95% CI (LL, UL)		
T0	69.18 ± 10.78	(65.55, 72.80)	71.44 ± 10.41	(67.94, 74.94)	−0.881	0.381
T1	83.03 ± 8.90	(80.04, 86.02)	72.94 ± 11.90	(68.81, 77.06)	3.916	< 0.001
T2	81.96 ± 9.87	(79.66, 86.52)	72.12 ± 11.46	(68.67, 76.82)	3.592	< 0.001
T3	80.45 ± 9.52	(77.40, 84.50)	71.38 ± 11.22	(64.54, 71.73)	3.418	< 0.001

*Note:* T0: baseline, T1: 1 month, T2: 3 months, T3: 6 months. T2: BBIP (*n* = 33) Routine care (*n* = 32); T3: BBIP (*n* = 32) Routine care (*n* = 31).

**FIGURE 2:**
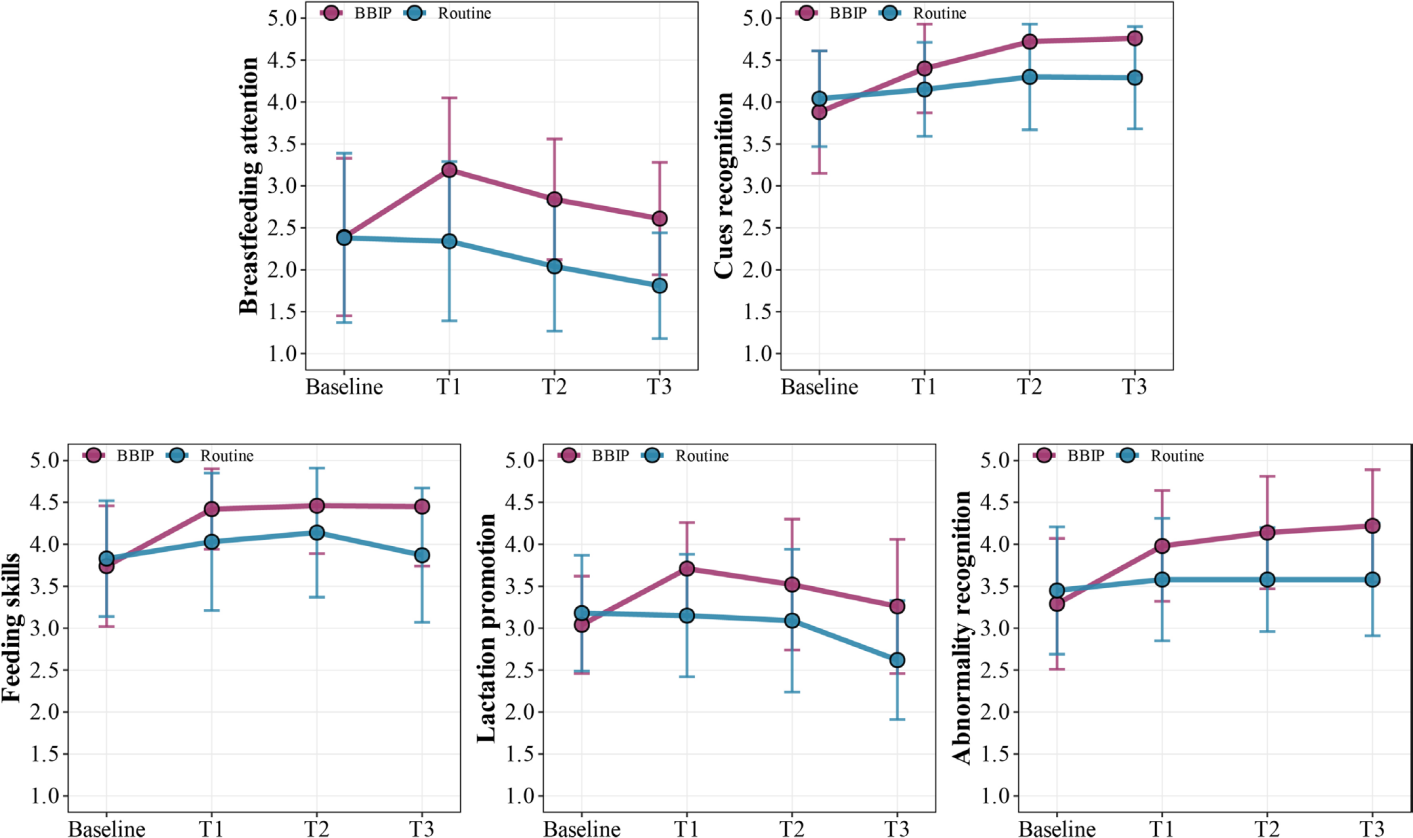
Comparison of scores across different dimensions between the two groups.

All dimensions showed significant improvement immediately post‐intervention in the BBIP group. Breastfeeding attention and lactation promotion scores demonstrated an initial increase followed by a gradual decline. Across the three dimensions: cue recognition, feeding skills, and abnormality recognition, the BBIP group consistently maintained superior scores, demonstrating statistically significant advantages over the control group throughout all time points (Figure 2).

### 3.3 Exclusive Breastfeeding Rates

The BBIP group showed significantly increased exclusive breastfeeding rates of 47.1%, 45.5%, and 43.8% at 1, 3, and 6 months post‐intervention. The proportion of breast milk in the daily diet within the BBIP group peaked at approximately 78.35% at 1 month of age and subsequently gradually declined to 56.40% by 6 months of age, remaining above the baseline level of 54.38%. In contrast, the routine care group showed a significant decline in the proportion of breast milk, decreasing from a baseline of 49.65% to 28.80% at 6 months. However, the proportion of breast milk in the daily diet declined over time in both groups (Table 3).

**TABLE 3:** Breastfeeding of CHD infants in the two groups at 1–6 months.

Time	Exclusive breastfeeding					Proportion of breast milk in daily diet				
	Routine care (*n* = 34)	BBIP (*n* = 34)	*p*			Routine care (*n* = 34)	BBIP (*n* = 34)	*p*		
			Intercept	Time	Group			Intercept	Time	Group
T0	4 (11.8)	8 (23.5)	< 0.001	0.066	0.037	49.65 ± 30.42	54.38 ± 34.11	< 0.001	0.027	0.012
T1	10 (29.4)	16 (47.1)				52.63 ± 42.30	78.35 ± 28.27			
T2	9 (28.1)	15 (45.5)				50.33 ± 43.89	69.67 ± 38.15			
T3	4 (12.9)	14 (43.8)				28.80 ± 37.22	56.40 ± 42.84			

*Note:* T2: BBIP (*n* = 33) Routine care (*n* = 32); T3: BBIP (*n* = 32) Routine care (*n* = 31).

### 3.4 The Growth Status of CHD Infants

At 6 months, the BBIP group showed significantly higher endpoint WAZ (*P* < 0.05), though endpoint LAZ did not differ significantly (*p* = 0.103). The Z‐scores revealed no significant differences between the two groups in either ΔWAZ (*p* = 0.825) or ΔLAZ (*p* = 0.324). These results demonstrate comparable improvements in weight gain and linear growth trajectories over the 6‐month intervention, supporting the non‐inferiority of BBIP to routine care in promoting growth for infants with CHD (Table 4).

**TABLE 4:** Comparison of WAZ and LAZ between two groups of infants.

Project	BBIP (*n* = 34)	Routine care (*n* = 34)	Test statistic	*p*
Baseline WAZ	0.26 ± 0.93	−0.11 ± 1.16	2.135	0.149
6 months WAZ	1.15 ± 0.78	0.66 ± 0.96	4.915	0.030
△WAZ	0.87 ± 0.92	0.82 ± 0.95	0.05	0.825
Baseline HAZ	0.23 ± 0.67	0.14 ± 0.74	0.284	0.596
6 months HAZ	0.87 ± 0.69	0.57 ± 0.74	2.735	0.103
△HAZ	0.64 ± 0.78	0.44 ± 0.83	0.99	0.324

*Note:* Six months: BBIP (*n* = 32) Routine care (*n* = 31).

## 4 Discussion

In this study, the BBIP intervention systematically addressed the key drivers of breastfeeding behavior within the BCW framework through a multidimensional, theory‐based approach.

### 4.1 The Key Role of Behavioral Intervention in Improving Breastfeeding Practices

Our findings suggest that this intervention significantly enhanced breastfeeding practices among CHD mothers, particularly during the early postpartum period. These results align with the review by Brockway et al. (2017), which emphasized the value of educational and support‐based interventions in enhancing breastfeeding self‐efficacy (BSE) and practices during early infancy.

The BBIP group maintained significantly higher scores than the control group across three key dimensions: cue recognition, breastfeeding technique, and abnormality identification. This sustained intergroup difference indicates that the behavioral intervention conferred durable efficacy in enhancing breastfeeding competencies. Specifically, hands‐on home‐visit guidance within BBIP reinforced CHD infants mothers' skills and safety awareness, with these effects remaining stable over time. Yu et al. (2021) similarly reported positive outcomes from WeChat‐based breastfeeding education among mothers of infants post‐cardiac surgery. Successful behavioral support should take cognizance of personal preferences, environment, and contextual factors (Reid et al. 2022). These findings support the potential of digital platforms to reinforce behavioral change in feeding practices.

### 4.2 Impact of Behavioral Intervention on Exclusive Breastfeeding Rates

Compared to the control group, mothers of infants with CHD in the BBIP group maintained consistently higher rates of exclusive breastfeeding at all follow‐up points. This finding aligns with improvements demonstrated by other structured breastfeeding support programs (Huang et al. 2019; Zielinska et al. 2017). Research indicates that exclusive breastfeeding for ≥ 14.9 weeks significantly increases the maintenance rate of any breastfeeding at 20 weeks (Dozier et al. 2018). While the intervention significantly increased EBF rates throughout the study period, the decline observed particularly at 6 months highlights the need for future interventions to be enhanced. This trend, consistent with global patterns, is primarily attributed to challenges associated with mothers' return to work and the introduction of complementary foods (Vilar‐Compte et al. 2021). Specifically, enhanced support for working mothers and specialized guidance on complementary feeding practices for infants with CHD are crucial to sustain exclusive breastfeeding for a longer duration.

### 4.3 Impact of Breastfeeding Promotion on CHD Infant Growth

Infants with CHD exhibit elevated energy demands (Haase et al. 2009; Mills et al. 2022). Conventional practice often considers exclusive breastfeeding insufficient to meet these heightened nutritional requirements. To address this challenge, the Breastfeeding Promotion Intervention Program (BBIP) implemented post‐feeding weight monitoring as a core strategy (Gregory 2018; Kent et al. 2015), enabling mothers to quantify infant breast milk intake accurately. Through structured WeChat follow‐ups to provide individualized guidance, this program enhances maternal engagement in nutritional management through real‐time feedback. This approach is grounded in behavioral theories that emphasize self‐monitoring and responsive caregiving (Peñacoba and Catala 2019; Wang et al. 2021). Critically, infants in the intervention group with higher breastfeeding exposure demonstrated non‐inferior growth outcomes compared to those with higher formula feeding reliance in the control group. These findings indicate that structured behavioral interventions enable breastfeeding to achieve growth outcomes comparable to formula feeding in mild CHD infants.

## 5 Conclusions

This study confirms that the BBIP constructed based on the BCW framework effectively improves breastfeeding practices through multidimensional intervention strategies. Breastfeeding achieved growth outcomes non‐inferior to formula‐dependent feeding in infants with mild CHD, which strongly contradicts prevailing clinical perceptions regarding the inadequacy of breastfeeding. Future research should evaluate the long‐term effects of breastfeeding on neurodevelopmental outcomes in infants with CHD and explore effective strategies for sustaining breastfeeding beyond 6 months of age.

## 6 Limitations

Some limitations of this study should be acknowledged. As a single‐center trial including only infants with mild CHD, the findings may lack generalizability to broader populations or complex cardiac conditions. The short‐term follow‐up period limited our ability to assess long‐term outcomes. In addition, self‐reported outcome measures tend to introduce an element of information bias, potentially affecting the accuracy of the data. As anthropometric measurements were conducted at infants' constituent community hospitals, potential inter‐facility variations constitute a limitation of this study.

## Author Contributions

**Huimei Wang:** methodology, investigation, formal analysis, project administration, visualization, writing – original draft. **Qi Zhang:** methodology, data curation, investigation, formal analysis, writing – original draft. **Yu Sun:** investigation. **Xueping Zhang:** investigation. **Yuehong Ren:** investigation. **Yalan Dou:** methodology, formal analysis. **Chuyi Zhan:** data curation, visualization. **Ming Ye:** resources, supervision validation, writing – review and editing. **Ying Gu:** conceptualization, funding acquisition, methodology, project administration, resources, supervision validation, writing – review and editing.

## Ethics Statement

No infants in either group received high‐energy fortified feedings.

## Conflicts of Interest

The authors declare no conflicts of interest.

## Supporting information


**Data S1:** fsn370907‐sup‐0001‐supinfo.zip.

## Data Availability

The original contributions presented in the study are included in the article and Supporting Information S1; further inquiries can be directed to the corresponding authors.
