# Supplementary material for: The Effect of Behavioral Intervention on Maternal Breastfeeding Practice and Infant Growth in Congenital Heart Disease: A Randomized Controlled Trial
Source: Food Sci Nutr. 2025 Sep 14;13(9):e70907. doi: 10.1002/fsn3.70907 (PMC12433894; doi:10.1002/fsn3.70907)
Supplement: Supplementary file 1 — Data S1: fsn370907‐sup‐0001‐supinfo.zip. [file FSN3-13-e70907-s001.zip › Breastfeeding Behavior Questionnaire for Mothers of Infants with Congenital Heart Disease.docx]

**Breastfeeding Behavior Questionnaire for Mothers of Infants with Congenital Heart Disease**

| **Dimensions** | **Item Content** | **Never** | **Occasionally** | **Sometimes** | **Often** | **Completely** |
| --- | --- | --- | --- | --- | --- | --- |
| I. Mothers' Concern for Breastfeeding in Infants with Congenital Heart Disease | 1.I will actively learn about breastfeeding in infants with congenital heart disease. | 1 | 2 | 3 | 4 | 5 |
|  | 2. I will actively record my baby's breastfeeding progress. | 1 | 2 | 3 | 4 | 5 |
|  | 3. I will measure my baby's weight before and after breastfeeding to assess breast milk intake. | 1 | 2 | 3 | 4 | 5 |
| II. Recognizing Infant Hunger and Fullness Cues | 1 .I judge hunger by my baby's facial expressions, movements, and sounds (crying, fussing, rooting, or thumb sucking). | 1 | 2 | 3 | 4 | 5 |
|  | 2. I judge fullness by my baby's movements (stopping sucking or spitting out the nipple) and feeding speed (slowing down feeding). | 1 | 2 | 3 | 4 | 5 |
|  | 3. I start breastfeeding when my baby shows rooting movements or thumb sucking. | 1 | 2 | 3 | 4 | 5 |
|  | 4. I stop breastfeeding when my baby slows down, falls asleep, stops sucking, or spits out the nipple. | 1 | 2 | 3 | 4 | 5 |
| III. Breastfeeding Techniques | 1. When breastfeeding, I choose a position that's comfortable for both me and my baby, keeping them close to me, their ears, shoulders, and hips aligned, and their neck naturally tilted forward or backward. | 1 | 2 | 3 | 4 | 5 |
|  | 2. When breastfeeding, I help my baby open their mouth wide to latch onto the nipple, covering most of the areola to maintain a good latch. | 1 | 2 | 3 | 4 | 5 |
|  | 3 .After feeding, I hold my baby upright and pat their back gently to reduce gas and prevent regurgitation and aspiration. | 1 | 2 | 3 | 4 | 5 |
|  | 4. I limit each feeding to under 30 minutes to avoid prolonged sucking that can put strain on the baby's heart. | 1 | 2 | 3 | 4 | 5 |
| IV. Methods to Promote Lactation | 1. Before my baby starts sucking, I apply a warm compress to my breast to promote blood circulation. | 1 | 2 | 3 | 4 | 5 |
|  | 2. I gently pat or massage my breast from the outer edge toward the areola to promote milk production. | 1 | 2 | 3 | 4 | 5 |
|  | 3. I alternate breastfeeding. | 1 | 2 | 3 | 4 | 5 |
|  | 4. I breastfeed on demand and empty my breasts after feedings. | 1 | 2 | 3 | 4 | 5 |
|  | 5. I ensure a sufficient daily supply of high-quality protein, such as milk, eggs, fish, and shrimp, as well as a diet rich in fresh fruits and vegetables and a healthy intake of water. | 1 | 2 | 3 | 4 | 5 |
|  | 6. After breastfeeding, I'll take advantage of the opportunity to get extra sleep while my baby is asleep to promote prolactin secretion. | 1 | 2 | 3 | 4 | 5 |
| V. Identifying and Addressing Choking During Breastfeeding in Babies with Congenital Heart Disease | 1. When my baby cries, I'll comfort them first, then start breastfeeding to prevent choking caused by crying. | 1 | 2 | 3 | 4 | 5 |
|  | 2. During breastfeeding, I'll squeeze my breasts to control the flow of milk. | 1 | 2 | 3 | 4 | 5 |
|  | 3 .If my baby is eager to feed, I'll let them take a few sips and then pause before feeding again. | 1 | 2 | 3 | 4 | 5 |
|  | 4 .If my baby chokes, I'll tilt their head to the side and pat their back to help release any remaining milk. | 1 | 2 | 3 | 4 | 5 |
