# Supplementary material for: The Effect of Behavioral Intervention on Maternal Breastfeeding Practice and Infant Growth in Congenital Heart Disease: A Randomized Controlled Trial
Source: Food Sci Nutr. 2025 Sep 14;13(9):e70907. doi: 10.1002/fsn3.70907 (PMC12433894; doi:10.1002/fsn3.70907)
Supplement: Supplementary file 1 — Data S1: fsn370907‐sup‐0001‐supinfo.zip. [file FSN3-13-e70907-s001.zip › Breastfeeding Home Visit Protocol.docx]

**Breastfeeding Home Visit Protocol**

**I．Pre-visit Preparation**

Contact the family in advance to confirm the visit time, and prepare hand disinfectant, mask, shoe covers, electronic scale, work ID and other items in the visit bag.

**II. Mother and infant assessment**

1. On-site assessment of the mother's sleep, nutrition, breastfeeding skills, etc.

2. Assess the baby's mental state, sleep, and measure the baby's weight and height.

**III．Breastfeeding Guidance**

1. Breastfeeding Behavior

(1) Posture: It is recommended that the mother breastfeeds in a sitting position. During breastfeeding, the baby faces the mother and stays close to the mother, with the baby's head and body in a straight line, the face close to the mother's breast, and the chin touching the breast. Be careful not to press the breast against the baby's nasal cavity to keep the airway open.

(2) Sucking: The mother gently touches the baby's mouth with the nipple to help the baby open his mouth and suck the nipple and most of the areola. When breastfeeding, the nipple should be placed deep in the baby's mouth, against the upper palate. If the mother finds that the baby is breathing rapidly and the sucking and swallowing are not coordinated, she can pause and pull the breast slightly outward to allow the baby to swallow the breast milk before continuing to feed to prevent choking or aspiration.

(3) Sucking: The mother can feel the baby sucking slowly and deeply.

(4) Swallowing: For effective breastfeeding, the mother can hear the baby swallowing.

2. Key points to observe during breastfeeding for CHD infants

(1) When feeding, if the baby eats quickly, you can pinch the breast with your hands to control the flow of breast milk.

(2) Feeding alternately from one breast to the other. Empty one breast and then observe whether the baby wants to continue eating. If not, empty the other breast. Whether the breast is empty or not is a matter of whether the supply and demand are balanced. If the milk production is low, the mother should try to feed and pump more frequently.

(3) Feeding time should be controlled within 30 minutes.

(4) Hold the baby upright and pat him gently after feeding.

(5) If the baby falls asleep during breastfeeding and then shows signs of continuing breastfeeding, do not use a formula bottle for the sake of convenience. Continue breastfeeding. The composition of foremilk and hindmilk is different and there is no clear time interval between them. Foremilk has a higher water content and hindmilk has a higher fat content, which promotes weight gain.

3. Calculation of the patient's milk intake during breastfeeding

(1) It is recommended that families prepare their own baby weight and height measuring device, with weight accurate to 0.01kg.

(2) The calculation formula is as follows:

Breast milk intake per feeding (ml)

= $\frac{\mathrm{Baby}'s weight after breastfeeding\left( g \right)- Baby’s weight before breastfeeding (g)}{Breast milk density (g/ml)}$

(3) Breast milk density is approximately 1.03 g/ml

(4) When measuring the baby's weight before and after a single breastfeeding, the baby scale should be zeroed in advance, and the patient's clothes and diapers should be removed. After measurement, record the weight in the "Breastfeeding Diary".

4. Precautions for breast pumping

(1) It is recommended that mothers express breast milk eight times a day to achieve a total of 500 ml of breast milk; if they express breast milk 10-12 times a day, they can achieve a total of 750 ml of breast milk.

(2) Preparation before breast pumping: Clean the surface of the breast pump and gently massage the breast. If milk leaks naturally, there is no need to massage the breast.

(3) Choose a suitable breast pump and pump under the maximum comfortable negative pressure.

(4) Express breast milk for 15-20 minutes. Wait 2 minutes after observing that the milk has stopped flowing, and then stop expressing.

(5) After expressing breast milk, write the date, time, and amount of milk on the bottle cap with a ballpoint pen. Do not exceed the scale line on the milk storage bottle to prevent the milk from spilling and contaminating the entire bottle. The milk storage bottle cap must be tightened and stored in a sealed environment. It is strictly forbidden to mix breast milk expressed at different times.

(6) Countermeasures for nipple pain: Apply sheep fat ointment or hydrogel. If a wound occurs, apply Bactroban and seek medical attention immediately.

5. Storage of breast milk and cleaning of utensils

(1) During the day, breast milk before 12 noon should be frozen and stored in the refrigerator after 12 noon.

(2) Store breast milk in a single compartment of the milk storage bottle. Do not mix with other foods. Do not place it on the shelf of the refrigerator door or store breast milk close to the refrigerator wall.

(3) Cleaning accessories: Wash hands with the seven-step hand washing method, disassemble accessories, and rinse off milk stains; use bottle cleaner and bottle brush to clean thoroughly; use microwave sterilizer bags and sterilizers to sterilize; boiling and scalding are not sterilization.

6. Common infections and breastfeeding guidance for mothers

(1) Direct breastfeeding is not recommended for mothers infected with the new coronavirus, but mothers of infected children are encouraged to separate mothers and babies and express breast milk for indirect breastfeeding via bottle by others.

(2) When mothers are infected with influenza virus, they should be isolated and avoid direct breastfeeding. Milk can be expressed and fed by others without sterilization. After the mother's symptoms disappear, she can breastfeed directly.

(3) Choice of anti-cold medicine: Acetaminophen is the mainstream recommendation, and ibuprofen-type drugs are still controversial.

1. Choice of analgesics: 1) NSAIDs: Acetaminophen, ibuprofen, and celecoxib are recommended; moderately safe: ketoprofen, naproxen, and diclofenac sodium; 2) Opioid analgesics: Generally not recommended. If necessary, fentanyl transdermal patches, morphine, tramadol, and codeine are options, but this is still controversial and must be selected by a clinical physician after weighing the pros and cons.

(5) When mothers suffer from mastitis or breast abscess, most of them can breastfeed, and emptying breast milk is an important treatment method.

(6) Mothers infected with hepatitis B virus can breastfeed. Mothers infected with hepatitis C virus can breastfeed. However, if the nipples are cracked or bleeding or the newborn has ulcers or lesions in the mouth, direct breastfeeding should be suspended.

(7) If the mother is infected with herpes simplex virus, if there is no herpes in the breast, direct breastfeeding can be done to avoid the baby from coming into contact with other herpes lesions; if there is herpes in the breast, the breast milk should be sterilized before feeding.

7. Nutritional components of breast milk at different stages

There is no significant difference in fat, water, energy, and density of breast milk at different ages, but the protein, lactose, and mineral content of breast milk at one month after delivery is higher than that of breast milk at two and three months. After 12 months of breastfeeding, mature breast milk does not lose its biologically active components and still has significant nutritional value.
