# Supplementary material for: The Effect of Behavioral Intervention on Maternal Breastfeeding Practice and Infant Growth in Congenital Heart Disease: A Randomized Controlled Trial
Source: Food Sci Nutr. 2025 Sep 14;13(9):e70907. doi: 10.1002/fsn3.70907 (PMC12433894; doi:10.1002/fsn3.70907)
Supplement: Supplementary file 1 — Data S1: fsn370907‐sup‐0001‐supinfo.zip. [file FSN3-13-e70907-s001.zip › The BCW framework implementation.docx]

BCW Framework Description:

The Behavior Change Wheel (BCW) theory was first developed by Michie in 2011 based on the integration of 19 behavior change intervention frameworks. It is a theory that can cover all intervention functions or policies. The BCW theory proposes that behavioral change requires the joint action of ability, opportunity and motivation, and that these three behavioral elements need to be improved through nine major intervention functions.

The whole classification system in terms of a ‘behaviour change wheel’ (BCW) with three layers as shown in the Figure below.


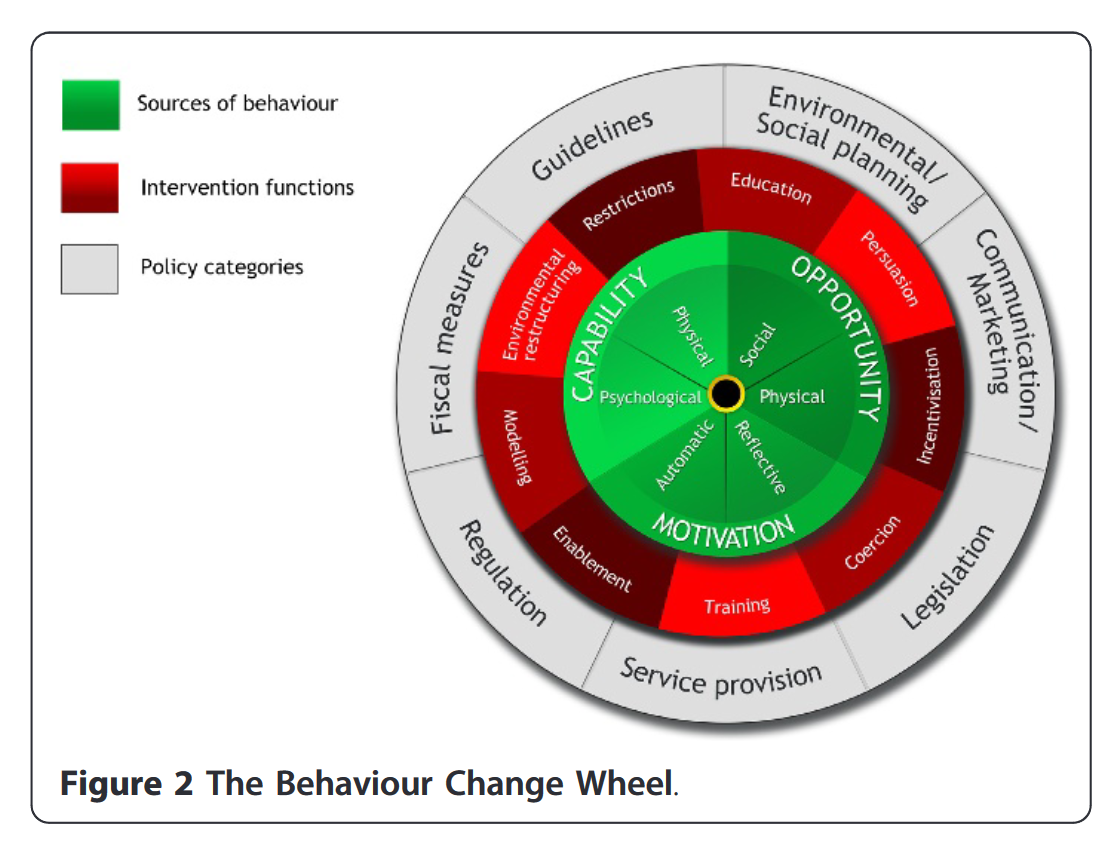


The sources of behaviour layer used the Capability, Opportunity, Motivation and Behavior model (COM-B model) to help identify the factors that influence individual behavior changes.

The intervention functions layer contains nine intervention functions, namely the means of behavior change, including education, persuasion, motivation, coercion, training, restriction, environmental reconstruction, modeling and implementation.

The policy categories layer consists of seven policy categories, including communication, guidelines, fiscal policy, management regulations, laws, environmental and social planning, and public services, which are mainly used to assist in the implementation of intervention functions.

This study was guided by the BCW theory, and took the ability, opportunity, and motivation to promote breastfeeding behavior of mothers of CHD infants as the core elements. (see table below)

| **Dimension** | **Intervention Contents** |
| --- | --- |
| **Capabilities** | Home Visit :Skills in breastfeeding support, including recognizing infant hunger cues, correcting latch-on positions, and demonstrating pre-/post-feed weighing methods. |
|  | Education Materials: Provision of science-based educational handouts and instructional videos. |
|  | Training on recognizing abnormal feeding situations in infants with CHD. |
| **Opportunities** | Social Support: Establishing support systems involving family members and professionals |
|  | Physical Environment:Creating conducive environments for breastfeeding support. |
|  | Information Resources:Conducting home visits.Offering online consultations.Distributing educational handouts. |
| **Motivation** | Measures to enhance maternal confidence and motivation include:  Emphasizing the benefits of breastfeeding across various settings.  Encouraging mothers to maintain feeding logs.  Providing ongoing online support from healthcare professionals. |
