# Supplementary material for: The Effect of Behavioral Intervention on Maternal Breastfeeding Practice and Infant Growth in Congenital Heart Disease: A Randomized Controlled Trial
Source: Food Sci Nutr. 2025 Sep 14;13(9):e70907. doi: 10.1002/fsn3.70907 (PMC12433894; doi:10.1002/fsn3.70907)
Supplement: Supplementary file 1 — Data S1: fsn370907‐sup‐0001‐supinfo.zip. [file FSN3-13-e70907-s001.zip › Training schedule.docx]

The training content for the research team and intervention nurses includes the background of the project, the implementation process of the intervention plan, ethical standards, home visit implementation process, data collection, and online question and answer sessions. It combines theoretical training with case practice. After the training, they are rquired to pass written tests and simulation assessments to ensure consistency in implementation.

| Content | Trainer | Training time | Assessment Method |
| --- | --- | --- | --- |
| Project Background | Gu Ying (PI) | 60 minutes | written |
| Implementation of project standardization process | Wang Huimei (Coordinator) | 120 minutes | written |
| Questionnaire Standardization Record | Wang Huimei (Coordinator) | 30 minutes | written |
| Breastfeeding of infants with congenital heart disease | Wang Huimei (Coordinator) | 60 minutes | written |
| Promotion of breastfeeding and management of related complications | Zhang Xueping (breastfeeding therapist) | 120 minutes | Written + Simulation |
